# Cost Effectiveness of Screening Colonoscopy Depends on Adequate Bowel Preparation Rates – A Modeling Study

**DOI:** 10.1371/journal.pone.0167452

**Published:** 2016-12-09

**Authors:** James Kingsley, Siddharth Karanth, Frances Lee Revere, Deepak Agrawal

**Affiliations:** 1 Department of Internal Medicine, Texas Health Presbyterian Hospital, Dallas, Texas, United States of America; 2 School of Public Health, University of Texas Health Science Center, Houston, Texas, United States of America; 3 Department of Internal Medicine, University of Texas Southwestern Medical Center, Dallas, Texas, United States of America; University Hospital Llandough, UNITED KINGDOM

## Abstract

**Background:**

Inadequate bowel preparation during screening colonoscopy necessitates repeating colonoscopy. Studies suggest inadequate bowel preparation rates of 20–60%. This increases the cost of colonoscopy for our society.

**Aim:**

The aim of this study is to determine the impact of inadequate bowel preparation rate on the cost effectiveness of colonoscopy compared to other screening strategies for colorectal cancer (CRC).

**Methods:**

A microsimulation model of CRC screening strategies for the general population at average risk for CRC. The strategies include fecal immunochemistry test (FIT) every year, colonoscopy every ten years, sigmoidoscopy every five years, or stool DNA test every 3 years. The screening could be performed at private practice offices, outpatient hospitals, and ambulatory surgical centers.

**Results:**

At the current assumed inadequate bowel preparation rate of 25%, the cost of colonoscopy as a screening strategy is above society’s willingness to pay (<$50,000/QALY). Threshold analysis demonstrated that an inadequate bowel preparation rate of 13% or less is necessary before colonoscopy is considered more cost effective than FIT. At inadequate bowel preparation rates of 25%, colonoscopy is still more cost effective compared to sigmoidoscopy and stool DNA test. Sensitivity analysis of all inputs adjusted by ±10% showed incremental cost effectiveness ratio values were influenced most by the specificity, adherence, and sensitivity of FIT and colonoscopy.

**Conclusions:**

Screening colonoscopy is not a cost effective strategy when compared with fecal immunochemical test, as long as the inadequate bowel preparation rate is greater than 13%.

## Introduction

Colonoscopy, sigmoidoscopy and stool tests for occult blood are recommended cost effective strategies for colorectal cancer (CRC) screening in the United States (US) [1]. The preferred method of CRC screening varies among different countries. Many countries have nationally organized screening programs that are centrally funded and controlled. These countries tend to favor stool tests. Other countries such as the US, have a more decentralized approach with multiple sources of funding and hence multiple modes of delivery, assessment and follow-up. In the US, screening colonoscopies are promoted as a preferred test. Despite significantly higher costs, colonoscopies are considered more cost-effective since they allow detection and removal of smaller precancerous polyps rather than simply early detection of cancer or in some cases advanced adenomas. The assumption in these analyses is that colonoscopies are performed and repeated per established guidelines i.e. if no polyp is detected (negative colonoscopy) screening colonoscopy is repeated in ten years, if adenomatous polyps are detected (positive colonoscopy) the surveillance colonoscopy is performed in three to five years [2]. However, in reality, colonoscopies are often repeated earlier than recommended intervals. Studies suggest colonoscopy overuse rates up to 25%[3–15], which is a significant problem, since it exposes patients to procedure risks without added benefit and is a poor use of resources.

Reasons for overuse of colonoscopies include endoscopists’ preferences [9–12] patients’ preferences [9–12] and inadequate bowel preparation [12–15]. Guidelines recommend repeating colonoscopy within a year, when bowel prep is inadequate due to concern about missing polyps. The reported rate of inadequate bowel preparations ranges from 5%-60%, with most studies citing 25%[3–15]. This wide range indicates that it is possible to address the variables leading to inadequate bowel preparations. However, this is often not prioritized due to logistical difficulties, resource investment and reimbursement pressures.

It is estimated that population-wide inadequate bowel preparation results in colonoscopy being repeated every 7.8 years in average risk patients instead of every ten years [16]. This increases the cost of colonoscopy as a population screening strategy and may even make screening colonoscopy a less cost-effective strategy when compared to less efficacious strategies. We performed a cost analysis of colorectal cancer (CRC) screening strategies incorporating inadequate bowel preparation rates to determine if colonoscopy is the most cost effective strategy.

## Methods

### Decision Model

**Fig 1:**
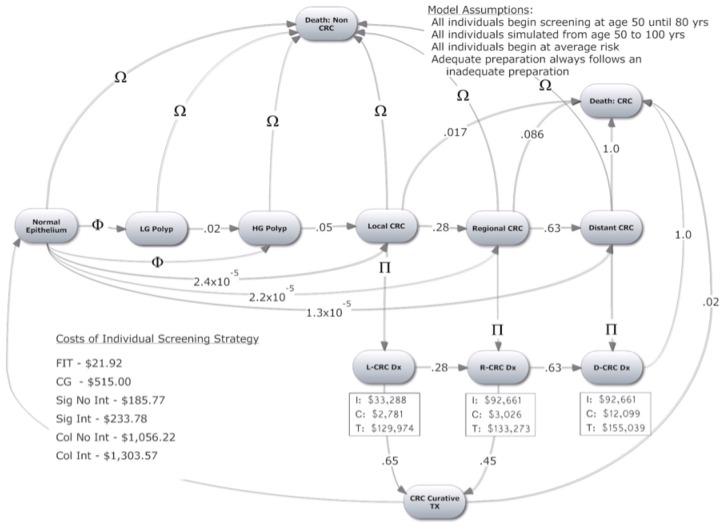
Markov Model. (Ω) varies by age according to US Actuarial Tables from 2014. (Φ) varies by risk factors: Age (0.011–0.019), History of High Grade Dysplasia (0.08), History of CRC (0.25). (Π) Diagnosis achieved through screening strategy (p value dependent on screening modality) & symptom presentation per year for total of 2 years (Local 0.22; Regional 0.40; Distant 1.00). (I) Costs of initial CRC care. (C) Costs of continual CRC care each year. (T) Costs of Terminal CRC care (End of Life). (LG) Low Grade. (HG) High Grade.

We constructed a Markov state transition model to examine the cost-effectiveness of four CRC screening strategies. The model is based on the natural history of CRC. The model tracks individual patients from age 50 until death or age 100. All screening strategies stop at age 80 because screening beyond this age is not routinely recommended. Each cycle in the model is one year long. Each patient has an inherent risk of developing an adenoma depending on age, sex, and history of high-grade polyp/CRC. The model tracks type of adenoma (low grade or high grade). High-grade adenomas are >1cm, three or more in number or with villous dysplastic features on histology. All other adenomas are considered low-grade. These characteristics determine the probability of adenoma being found by a screening strategy and the risk of developing CRC, which progresses through stages: local, regional and distant colorectal cancer. Probability of survival after diagnosis of CRC depends on the stage of disease. Fig 1 depicts our model and possible transitions from one state to another. Screening strategies are superimposed on the model. The effectiveness of a screening strategy is determined by the test's ability to detect adenomas or CRC. All key assumptions used in the model are listed in Table 1.

**Table 1:** Model Inputs: Case Base Estimates Used in the Model.

Input Names	Base Case Estimate				Reference Used [Additional Support]
	FIT	Col	SDNA	Sig	
Sensitivity Low Grade Polyp	0.1	0.85	0.172	0.85	[53] [2,19,22,23,54–64]
Sensitivity High Grade Polyp	0.24	0.9	0.692	0.9	[53] [2,19,22,23,54–64]
Sensitivity Colorectal Cancer	0.7	0.95	0.923	0.95	[53] [2,19,22,23,54–64]
Specificity	0.95	0.95	0.866	0.92	[53] [2,19,22,23,54–64]
Percent of polyps within reach of Endoscopy [regardless of whether strategy successfully detects polyp or not]	-	100%	-	60%	[21] [2,22,23,35,55–63]
Inadequate Bowel Preparation Rate	0.25				[33] [3–15]
Probability that Inadequate Bowel Preparation is Poor Prep	0.29				[3] [57–64]
Probability that Inadequate Bowel Preparation is Fair Prep	0.71				[3] [57–64]
CRC Screening Adherence: % of population who are completely Up to Date	0.651				[29] [20,22]
CRC Screening Adherence: % of population who have never been screened.	0.277				[29] [20,22]
CRC Screening Adherence: % of population who have been screened but are not up to date	0.072				[29] [20,22]
Surveillance Adherence for those Completely Up to Date	0.95				[19] [3,20,22]
Surveillance Adherence for those Incompletely Up to Date	0.85				[19] [3,20,22]
Surveillance Adherence for those Never Screened	0.75				[19] [3,20,22]
Adherence to Instructions of Colonoscopy	0.53				[20] [2,21]
Adherence to Instructions of FIT or SDNA	0.8	-	0.8	-	[20] [2,21]
Follow Up Adherence with Colonoscopy After + FIT or SDNA	0.83	-	0.83	0.83	[20] [2,3,22]
Health Utility Weight for Initial CRC	0.9				[19] [21]
Health Utility Weight for Regional CRC	0.8				[19] [21]
Health Utility Weight for Distant CRC	0.76				[19][21]
Probability of Local Cancer Diagnosis	0.4				[57–64]
Probability of Regional Cancer Diagnosis	0.37				[57–64]
Probability of Distant Cancer Diagnosis	0.23				[57–64]
Probability of Local Cancer Cure	0.65				[57–64]
Probability of Regional Cancer Cure	0.45				[57–64]
Probability of Distant Cancer Cure	0				[57–64]
CRC Treatment Mortality	0.02				[21][57–64]
Complication Rate per Screening Event	-	0.0016	-	0.00016	[65][21,26,33,57–64]
Mortality Rate per Screening Event	-	7.4x10^-5^	-	-	[65] [21,26,33,57–64]
Prevalence of Polyp at First Screen	0.25				[66] [21,57–64]
Incidence of Low Grade Polyp without history of polyps or CRC	.011−.019*				[66] [21,57–64]
Incidence of High Grade Polyp without history of polyps or CRC	7.5x10^-4^				[66] [21,57–64]
Incidence of Low & High Grade Polyp with history of polyps but not CRC	0.08				[66] [21,57–64]
Incidence of Low & High Grade Polyp with history of CRC	0.25				[66] [21,57–64]
Ratio of Polyp Incidence that are LG vs. HG < 65yrs/old	0.95				[66] [21,57–64]
Ratio of Polyp Incidence that are LG vs. HG > 65yrs/old	0.679				[66] [21,57–64]
Annual Transition Probability from: LG Polyp—> HG Polyp	0.02				[21] [57–64]
Annual Transition Probability from: LG Polyp—> Local CRC Polyp	0.00697				[21] [57–64]
Annual Transition Probability from: HG Polyp—> Local CRC	0.05				[21] [57–64]
Annual Transition Probability from: Local CRC—> Regional CRC	0.28				[21] [57–64]
Annual Transition Probability from: Regional CRC—> Distant CRC	0.63				[21] [57–64]
Probability of Local Cancer Presentation each year for 2 years	0.22				[21] [57–64]
Probability of Regional Cancer Presentation each year for 2 years	0.4				[21] [57–64]
Probability of Distant Cancer Presentation over the course of 2 years	1				[21] [57–64]
Probability of Colorectal Cancer Occurring without Polyp each year	6.0x10^-5^				[57–64]
Probability of Local CRC from Normal Epithelium	0.68	0.56	0.68	0.53	[21] [57–64]
Probability of Regional from Normal Epithelium	0.22	0.32	0.22	0.33	[21] [57–64]
Probability of Distant CRC from Normal Epithelium	0.1	0.12	0.1	0.14	[21] [57–64]
Mortality Rate for Local Cancer each year for 5 years	0.0174				[20, 58–64]
Mortality Rate for Regional Cancer each year for 5 years	0.086				[20, 58–64]
Societal Willingness to Pay for 1 Additional QALY	$50,000.00				[15] [25]
Discount Rate Used for Costs & Effectiveness	3%				[15] [20,25,58–64]

CRC, colorectal cancer; LGD, low-grade dysplasia; HGD, high grade dysplasia; QALY, quality adjusted life years

* varies with age

The incidence of adenomatous polyps and colorectal cancer was determined from age-specific (per year [prevalence rates at autopsy from years 1970 to 1990 reported in the Surveillance, Epidemiology, and End Results (SEER) program data [17]. These decades were chosen because population screening or surveillance was not widespread enough to skew the natural history model. If a person develops CRC he/she carries a new yearly probability rate of mortality based on five-year survival rates and average length of survival by stage of cancer and age according to data from SEER [17,18]. To estimate the rates for death from causes other than CRC we used data from the USA Official Social Security Office’s Actuarial Life Tables of 2011.

Initial, regional and distant CRC were assigned health utility weights for 0.9, 0.8 and 0.76 respectively [19].

### Screening Strategies

The Markov model assigns patients to undergo one of the following recommended screening strategies: annual fecal immunochemical testing (FIT), flexible sigmoidoscopy (FS) every five years, colonoscopy every ten years and stool DNA test every three years. The characteristics of different screening tests were determined from independent review of literature (Table 1). Each screening strategy was assigned a different adherence rate based on published literature [20–22]. Patients undergoing colonoscopy could have an adequate or an inadequate prep. If the preparation was inadequate, colonoscopy was repeated at varying intervals based on averaged real world data [3]. Simulated patients with a positive stool test or polyps on sigmoidoscopy received a colonoscopy that same year. For the probability that the stool test was false positive, the patient underwent a negative colonoscopy, and then resumed with the primary screening strategy. If polyps were found, they were removed and patients were switched to surveillance colonoscopy in 3 years (high-grade adenomas) or 5 years (low-grade adenomas). We assumed that 95% of all colonoscopies reached the cecum [23].

### Adherence Rates

Most prior cost-effective studies assumed a 100% adherence rate for different screening strategies [24–27]. In reality, the adherence rates are much lower and differ among strategies. Since screening strategies vary considerably in costs, adherence rates can have large influence cost-effectiveness ratios. Non-adherence to subsequent screenings even within the same strategy can significantly affect the cost-effectiveness. However, even with a relatively a small degree of adherence with subsequent screenings, cost-effectiveness can be comparable to full participation [24–28].

We used reported adherence rates for US population and accordingly divided the population into three groups. The adherence rate was broken down into two gates of adherence. The first gate was whether the patient received a referral/order for a screening test. The second gate was whether or not the patient actually adhered to the recommendations. 27% of patients were never recommended CRC screening, 8% received recommendation once in their lifetime and 65% received recommendations consistently according to guidelines [27]. The second gate evaluated whether or not patients adhered to the recommendations (of note, this doesn’t include inadequate bowel preparations). Adherence rates varied according to type of screening strategy with less adherence for endoscopic procedures (52%) versus stool tests (92%) [20]. Thus, the relative end of life adherence rate was 38% for colonoscopy and sigmoidoscopy and 67% for fecal exams. Patients with positive stool tests were assumed to be 83% adherent with follow-up colonoscopy [20]. In addition, while non-adherers did not receive screening, if they were diagnosed with CRC by symptom presentation, and received curative treatment, they entered a surveillance regime, with a 75% adherence rate [19,20]. Alternative adherence assumptions, including 100% adherence, were explored in a sensitivity analysis.

### Inadequate Bowel Preparation

The definition of inadequate bowel preparation and the timing for repeat colonoscopy varies in both the theoretical realm and in practice. For this analysis, inadequate preparation was defined as those that result in a repeat colonoscopy earlier than the recommended interval for an inadequately prepared colon. Studies suggest that most gastroenterologists would repeat colonoscopies for intermediate (fair) and poor quality preps due to the possibility of missing polyps greater than five millimeter in size [20–32].

In our analysis we varied the inadequate bowel preparation rate using a threshold analysis, beginning at an ideal rate of 0% and ending at 30%. This range was based on the assumed current inadequate bowel preparation rate of 25% [3–15]. It was assumed that all patients with inadequate bowel preparations underwent repeat colonoscopy at the rates of 9% within one year, 5% in one to two years, 3% in two to three years and 83% in three to 10 years [14]. Notably, the inadequate bowel preparation rate in the model had no influence on the effectiveness portion of the cost-effectiveness calculation as all patients were rescheduled for colonoscopy.

### Cost of Screening Strategies and CRC Treatment

The calculation of costs in this study is notable for a number of reasons. First, a weighted average of Medicare and private insurance reimbursements was used for colonoscopies performed in an office setting, ambulatory surgical centers and hospital based endoscopy units. This is different from most prior studies on cost effectiveness, which calculated colonoscopy costs based only on Medicare payment rates. Medicare payment rate calculations result in underestimation of total costs since Medicare accounts for only 31% of screening colonoscopies and private insurance pays 1.5 to 2 times more than Medicare [33]. Furthermore, the reimbursement for screening colonoscopies performed at hospital based endoscopy units can be up to 30% higher [34]. The second notable cost calculation is the incorporation of the cost of sedation provided by anesthesiologists. The study assumed that the sedation in 30% of colonoscopies was administered by an anesthesiologist, although much higher numbers have been reported. Third, costs of lost wages and benefits due to the procedure were added, based on Bureau of Labor Statistics. Additionally, the cost for a follow-up colonoscopy was included for the fecal exams and the sigmoidoscopy that were positive (both true positives and false positives). The baseline costs used for our analysis are given in the Fig 1.

Net costs of CRC treatment from year 2009 were determined for each local, regional and advanced stage of cancer. These costs differed for the initial, continuing and terminal phases of care. All cost data associated with screening and treatment were adjusted to the real value of US dollar in 2014 using the Consumer Price Index for Medical Care. The costs of lost wages and benefits were derived from the Bureau of Labor Statistics and adjusted to 2014 US dollars using the Consumer Price Index.

### Methods of Model Analysis

The cost-effectiveness of different CRC screening strategies was estimated using a societal perspective.

Given the inherent limitations of ratios in cost-effectiveness (C/E), Net Monetary Benefit (NMB) was determined to allow for a more transparent comparison of multiple strategies. The NMB uses society’s willingness to pay for a quality adjusted life year (QALY) to convert the comparisons between strategies from ratios to arithmetic differences. Because NMB doesn’t utilize ratios, effectiveness calculations are not in the denominator and thus NMB is a better comparative measure when differences in effectiveness between strategies are small [34]. NMB was calculated by assuming a willingness to pay (WTP) of $50,000 per QALY gained. Costs and QALYs were discounted at an annual rate of 3%. The model was analyzed using TreeAge Pro 2015 (TreeAge Software Inc., Williamston, MA).

The analysis included 1-way and probabilistic sensitivity analysis (PSA). One-way sensitivity analysis was performed on key variables identified by a tornado diagram. For probabilistic sensitivity analysis- 100,000 individuals walked through a second order simulation and each individual’s probability (in second order) was the composite mean of 10,000 individuals walking through a first order simulation. The PSA assumed a uniform distribution for the probabilities, and a beta distribution for the QALYs in the model.

### Model Validation

External validation was performed by comparing our model’s outputs with those of well-established CEA models. For example, CEA performed by Sharaf et al [21] comparing FOBT, sigmoidoscopy and colonoscopy. The lifetime cost of colonoscopy per person in their model was $2564 compared to $2430 in our model. The Incremental Cost effective ratio (ICER) values for colonoscopy versus FIT reported by them were also similar to our model.

We also compared intermediate outputs within our model with the National Polyp Study and other well-established epidemiological data produced by the American Cancer Society and the National Cancer Institute. In our model the current utilization rates for each strategy showed a 0.0593 lifetime CRC incidence rate compared to 0.05 published by the American Cancer Society [35]. Our natural history model, on which the screening strategies were superimposed, had a standardized incidence based CRC mortality ratio of 0.40, which is comparable to the rate of 0.47 (95% CI .26–.80) published by the National Polyp Study [22]. Our standardized incidence based lifetime CRC incident ratio was 0.3989 compared to the National Polyp Study rate of 0.24 (95% CI .08–.56) [22]. Our model’s output for CRC mortality reduction with colonoscopies was 60%, which is comparable to the National Cancer Institute’s estimate of 60–70% mortality reduction [35].

## Results

The base case scenario in our model was designed to reflect real-life CRC screening patterns in the US, which show an inadequate preparation rate of 25% and variation in adherence rates for different screening strategies.

### Comparison of Screening Strategies vs. No Screening

The cost and effectiveness of different screening strategies are given in Table 2. Compared to no screening, FIT is the most cost effective (in terms of quality adjusted life year, QALY) screening strategy followed by colonoscopy, stool DNA and sigmoidoscopy. The cost of FIT ($1,579) is the least and stool DNA ($3,526) the most expensive. Only FIT and sigmoidoscopy are both more effective and less costly (dominant) compared with no screening. Colonoscopy cost $3,257 more per quality adjusted life year and stool DNA test cost $15,762 more per quality adjusted life year when compared to no screening strategy.

**Table 2:** CEA & ICER Results at 25% Inadequate Bowel Prep Rate for a cohort of 100,000 average-risk US citizens age 50 to 100yrs.

		FIT	Sigmoidoscopy	No Screening	Colonoscopy 25%	Stool DNA
CEA	Cost	$1578.73	$1914.09	$2103.12	$2429.81	$3526.45
	Effect	19.5057	19.4787	19.4168	19.5171	19.5071
	NMB*	$973706.27	$972020.91	$968736.88	$973425.19	$971246.00
ICER	FIT	-	Sig is Dominated	NS is Dominated	$74656.14	$1391228.57
	Sigmoidoscopy	-	-	NS is Dominated	$13430.21	$56773.24
	No Screening	-	-	-	$3257.13	$15762.24
	Colonoscopy 25%	-	-	-	-	SDNA is Dominated
	Stool DNA	-	-	-	-	-

Abbreviations: CEA, Cost Effectiveness Analysis; ICER, incremental cost effectiveness ratio; FIT, fecal immunoassay test; NS, No Screening; NMB, Net Monetary Benefit; SDNA, Stool DNA.

* At a Willingness to Pay for 1 Quality Adjusted Life Year of $50,000.

### Comparison of Screening Strategies with One Another

In incremental comparisons between each strategy (Table 2), colonoscopy at 25% inadequate preparation rate cost $74,656 per QALY more compared to FIT and hence was not cost-effective according to societies’ established willingness to pay (WTP) threshold for a single quality adjusted life year of $50,000. However, colonoscopy was more cost-effective compared to sigmoidoscopy and stool DNA. Stool DNA test was dominated as it was more costly and less effective than colonoscopy. Similarly the incremental cost effectiveness ratios of stool DNA compared with FIT and sigmoidoscopy were both above the WTP of $50,000. The net monetary benefit at WTP of $50,000 showed FIT to have more benefit than colonoscopy followed by sigmoidoscopy and stool DNA test.

### Comparison of Different Inadequate Preparation Rates

The cost-effectiveness and NMB of screening strategies were determined at different inadequate bowel preparation rates using a threshold analysis from 0% to 30% (Table 3). Colonoscopy was more cost effective compared to FIT (i.e. ICER < $50,000) when inadequate bowel preparation rates were 13% or lower. At inadequate preparation rate of 13%, incremental cost effectiveness ratio of colonoscopy compared with FIT was at societies willingness to pay threshold of $50,000. This suggests that 13% inadequate preparation rate is the threshold above which FIT becomes the preferred screening strategy. Colonoscopy remained a preferred screening strategy over sigmoidoscopy even at 30% inadequate preparation rate. Colonoscopy dominated stool DNA test at all inadequate preparation rates.

**Table 3:** Incremental Cost Effectiveness Ratios comparing colonoscopy at various bowel prep rates with other screening strategies.

Inadequate Preparation Rate	Cost	FIT	Sigmoidoscopy	No Screening	Stool DNA (SDNA)
**0%**	$1,982.50	$35,418.42	$1,781.51	NS is Dominated	SDNA is Dominated
**5%**	$2,043.70	$40,786.84	$3,375.26	NS is Dominated	SDNA is Dominated
**10%**	$2,102.86	$45,976.32	$4,915.89	NS is Dominated	SDNA is Dominated
**15%**	$2,181.05	$52,835.09	$6,952.08	$776.97	SDNA is Dominated
**20%**	$2,241.03	$58,096.49	$8,514.06	$1,374.98	SDNA is Dominated
**25%**	$2,429.81	$74,656.14	$13,430.21	$3,257.13	SDNA is Dominated
**30%**	$2,515.11	$82,138.60	$15,651.56	$4,107.58	SDNA is Dominated

### Sensitivity Analysis

One-way sensitivity analysis was performed on all inputs by increasing and decreasing the variables within 10% of the base case values to ensure inclusion of all the values reported in literature. The base results remained robust to the majority of the sensitivity analyses (Table 4). The incremental values for FIT and colonoscopy (25% inadequate preparation rate) are reported because they had the highest NMB compared to other tests. Specificity of FIT was the most influential variable; however, this is of limited practical significance as most studies have confirmed specificity of FIT for colon cancer to be 91–93%. The sensitivity of FIT had much less influence on the results. Provider reimbursement for a colonoscopy had a negligible impact on the results.

**Table 4:** Sensitivity Analysis.

Variables*	Variable Range Input	Low Value	High Value	Risk Percent
Specificity of FIT	0.855 to 0.997	16,006.3790	96,141.9481	45.14%
Adherence to FIT	0.72 to 0.88	59,286.6552	110,129.2013	18.17%
Colonoscopy Sensitivity for LG Polyp	0.765 to 0.935	60,225.2048	99,345.4563	10.76%
Adherence of Colonoscopy	0.586 to 0.716	66,029.7035	104,194.2306	10.24%
Colonoscopy Sensitivity for HG Polyp	0.81 to 0.99	56,133.4962	89,003.9174	7.60%
FIT Sensitivity for LG Polyp	0.09 to 0.11	65,414.5932	84,185.2703	2.48%
FIT Sensitivity for HG Polyp	0.216 to 0.264	63,230.9017	81,665.0094	2.39%
FIT Sensitivity for Colorectal Cancer	0.63 to 0.77	66,830.9051	83,756.4146	2.01%
Colonoscopy Sensitivity for Colorectal Cancer	0.855 to 0.997	71,987.1885	85,105.9302	1.21%
Cost of FIT	$18 to $26	74,043.6500	75,342.3300	< 1%
Cost of Colonoscopy (with Intervention)	$900 to $1,200	74,309.7600	74,963.5435	< 1%
Cost of Colonoscopy (without Intervention)	$1,100 to $1,500	74,604.7600	74,724.9900	< 1%
Cost of Initial Colon Cancer Treatment at a Stage of Diagnosis Specific Rate	+ 25%	-	69,333.9211	-

FIT, Fecal Immunochemical Test; LG, Low Grade; HG, High Grade

* Variables were chosen based on the results of a tornado diagram in order of highest ICER variance.

Stool DNA is a newly approved test, which is generally considered to be more effective than FIT but is significantly more expensive. Threshold analysis (at WTP of $50,000) show stool DNA test reimbursement would have to be $75 for it to be the preferred screening strategy.

**Fig 2:**
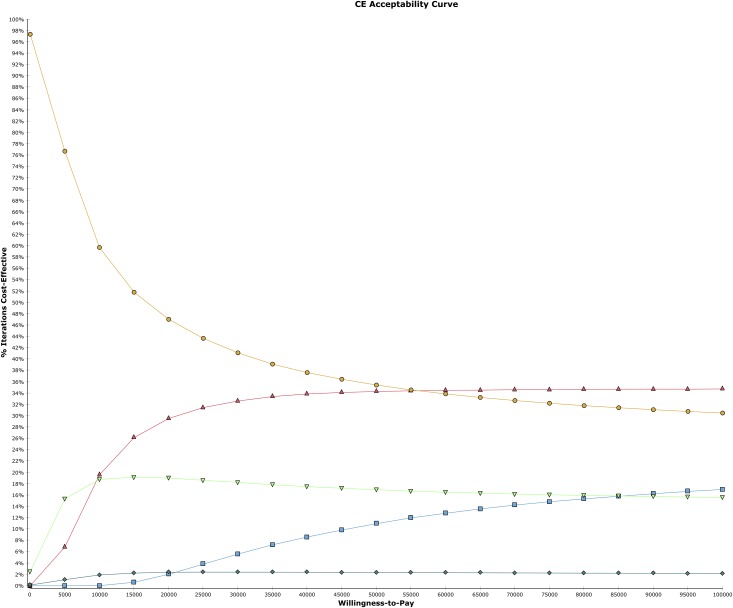
Cost Effectiveness Acceptability Curve. DNA Stool Test (blue square). Colonoscopy (red triangle). FIT (yellow circle). Sigmoidoscopy (green triangle). Natural History (turquoise diamond).

The cost-effectiveness acceptability curves (CEAC) from probabilistic sensitivity analysis were determined. Fig 2 shows the probability, based on the proportion of simulations in which a given CRC screening strategy is most cost effective for a range of maximum WTP thresholds. FIT is the most cost-effective strategy at WTP of $50,000 according to 36% of 10,000 model iterations. Colonoscopy (25% inadequate bowel preparation rate) becomes cost effective as WTP increases beyond $55,000 (in 35% iterations). Sigmoidoscopy is cost effective in less than 19% iterations and decreases slightly as the WTP increases beyond $55,000. Stool DNA test is not cost effective compared to any screening strategy except at WTP of $85,000 at which point it becomes more cost effective than only sigmoidoscopy.

## Discussion

Inadequate bowel preparation is defined as the inability to identify lesions larger than five millimeters [36,37]. However, judging bowel preparation as inadequate remains inconsistent and subjective. Guidelines suggest repeating colonoscopy for inadequate preparations, but they do not make a distinction between grades of inadequacy (intermediate or poor) [22]. The timing of repeat colonoscopy is left to the endoscopists’ discretion and thus highly variable, with most recommending shorter intervals as preparation worsens [20]. While colonoscopy has been shown to be cost-effective for colon cancer screening, the research does not take into account the cost of repeat colonoscopies due to inadequate preps. This analysis shows that screening colonoscopy is not a cost-effective strategy if more than 13% of colonoscopies are repeated because of inadequate bowel preps. These findings are more pronounced given the model assumed the current high costs of CRC treatment, which yields a cost-effectiveness advantage for colonoscopy because it can prevent, not simply detect, cancer.

Our analysis has several strengths. First, to calculate the cost of colonoscopy we used weighted averages of reimbursement by different third party payers. This best reflects, settings where colonoscopies are performed and the costs of anesthesia. Most cost-effective analyses use only Medicare reimbursement rates and thus underestimate the true cost burden of colonoscopies. Second, our model accounts for population adherence to screening across all strategies. This has been an important limitation of prior analyses because studies have shown higher adherence rates for stool tests compared to endoscopies. In fact, this can influence the outcomes of incremental cost effectiveness ratios. For example, in the cost-effective analysis of CRC screening strategies by Sharraf et al, FIT dominated colonoscopy since it was less expensive and more effective [21]. In our model, colonoscopy was more effective because FIT’s effectiveness is largely dependent on yearly adherence to avoid the emergence of CRC given its lower negative predictive rate. FIT has a poor sensitivity for dysplastic polyps, but this poor performance is made up by yearly exams. In a model, such as ours, where adherence to FIT is lower than 100%, the effectiveness of FIT would expectedly be lower. In fact, Sharaf et al demonstrated this point in a sensitivity analysis, which showed that as adherence rates for FIT were decreased toward real world rates, colonoscopy became increasingly cost effective [21]. Third, the recently approved stool DNA test was included as a screening strategy, using newly published data.

The model and analyses are based on a few significant assumptions. First, it was assumed that colonoscopies are repeated in all patients with inadequate bowel preparations. This assumption is based on both guidelines that recommend a repeat colonoscopy and the standard practice among gastroenterologists [30,31]. However, not all patients who are recommended repeat colonoscopy may get it. A study analyzing commercial and Medicare claims data found inadequate bowel preparation in 15%- 25% patients, but lower rates of repeat colonoscopies. The second assumption was colonoscopies with inadequate preparations were reimbursed at the same rate as those with adequate preparations. Medicare pays for a screening colonoscopy (without polyps) only once every ten years unless the physician informs Medicare that the colonoscopy was incomplete, by using billing modifier 53 [38]. Medicare then pays for the incomplete colonoscopy at half the rate of a regular colonoscopy. However, there is ambiguity around this billing practice and the billing modifier is rarely used. Medicare Part B claims data from 2009–2013 shows that only 2–3% of all colonoscopies were billed with modifier 53 [38]. A study of sample Medicare screening colonoscopies from 2000–2008 showed that of all the claims for colonoscopies without clear indications, only 2% were denied reimbursement by Medicare [39]. Hence, we feel that using full reimbursement rates for inadequate colonoscopies is justified. Third, it was assumed that all colonoscopies where a polyp was removed were adequate. In reality, it is not uncommon for an endoscopist to remove a small polyp when the preparation is intermediate and have the patient follow-up earlier than the recommended three to five years. Studies suggest that in 25–50% of patients with small adenomas, surveillance colonoscopies are recommended earlier than recommended in guidelines. These patients were more likely to have less than excellent preps. Thus, this analysis underestimates the burden of overuse of surveillance colonoscopy. The final assumption presumed patients follow only one screening strategy. In reality, patients may change between different strategies, but no data exists to quantify this potential and the purpose of the study was to compare one strategy to the other.

Our study has important clinical, financial and policy implications. Costs of screening colonoscopy have been under scrutiny in the last few years by third party payers and lay media. The increasing costs have prompted Medicare to propose more cuts in reimbursement rates for colonoscopy. This analysis shows that decreasing reimbursements would have minimal impact on overall costs. The professional societies fear that the cuts may even negatively impact the colonoscopy screening rates [40]. The approach instead should be to promote more effective use of screening colonoscopies, such as improving the inadequate bowel preparation rates and decreasing the costs associated with repeat colonoscopies. Screening colonoscopies are cost-effective if the inadequate bowel preparation rate is less than 13%; a rate that is certainly an achievable target with concerted efforts. The European Society of Gastroenterology [8] has a recommendation of inadequate bowel preparation rate of less than 10% while the American College of Gastroenterology [41] recently set this target as less than 15% for outpatients. Furthermore, the treatment arms of many randomized control trials had inadequate bowel preparation rates of 4–10% [42–48].

There are many variables that determine the quality of colon preparation. These include: patient related variables (medical comorbidities, demographics, socio-economic, literacy); preparation related factors (type, dose, timing); system related constraints (quality of preparation instructions, scheduling) and endoscopist related patterns of care (subjective rating of quality of colon preparation and time spent to clean the colon). Most of the factors that determine colon preparation quality can be controlled. For example, Hassan et al proposed a predictive model based on patients’ demographics and comorbidities that could theoretically decrease the inadequate preparation rate from 33% to 13% [48]. In practice, these proven interventions are infrequently adopted and most endoscopy units still prescribe a standard bowel preparation to all patients. This is likely because individualizing bowel preps is resource intensive and many institutions may not feel necessary to invest in these resources.

Based on these findings, perhaps inadequate bowel preparation rate should be a reportable quality indicator. After all, better quality of bowel preparation is linked to higher adenoma detection rate (ADR), which is now the most recognized and promoted quality indicator of screening colonoscopy. Proper bowel preparation provides significant additive value to ADR. For example, a polyp may be removed from a colon with an inadequate preparation counting towards ADR, but there may be other undetected polyps. Thus, total adenoma detection may be lower with intermediate versus high-quality bowel preparation, even as ADRs remain equivalent [49,50]. Lower inadequate bowel preparation rates would result in fewer repeat colonoscopies. This data could be used by third party payers, accountable care organizations and primary care physicians to guide their patients to endoscopy units that consistently have better preparation rates. It is likely that inadequate bowel preparation rates as a reportable metric would be an incentive for institutions to improve. It may also be an impetus for the endoscopists to try harder to wash some of the intermediate preps converting them to adequate. In a study comparing a public and private hospital affiliated to the same university, patients at the public hospital were more likely to have a repeat colonoscopy for imperfect preps (20% vs. 12.5%) and the endoscopists spent less time in washing the colon (7.5% vs. 10.3% of the total procedure time) [14]. Finally, one could argue that endoscopy units or institutions that consistently fail to improve their inadequate bowel preparation rates to less than 13% should offer FIT instead of colonoscopy as the preferred CRC screening strategy.

There has been much emphasis and resources spent on increasing CRC screening rates to “80% by 2018” [51,52]. This is certainly a worthy goal and it is estimated that increasing CRC rates from 60% to 80% could decrease CRC mortality by almost 33%. If this increase in CRC screening comes from increasing colonoscopy screening rates, it will cost significantly more with an inadequate bowel preparation rate of 25% compared to 13%. In fact, if the current inadequate preparation rates cannot be improved, then it is more cost effective to increase rates of FIT since 100% adherence rate with FIT offers more QALYs at a lesser cost compared to even 80% colonoscopy adherence rates.

Stool DNA test was compared with other screening strategies and found to be more cost effective than no screening but not compared to other screening strategies. This analysis determined $78 to be the threshold cost for stool DNA test to be cost effective. These results are similar to the cost effectiveness study performed by the Agency of Healthcare Research and Quality that calculated a cost of $34 -$60 for stool test to be a non-dominated option [40]. The slight difference in results is likely due to new data on cost and efficacy.

There are some limitations of the study, apart from those due to the aforementioned model assumptions. First, while the best available data were used for estimates, the source of data ranged from randomized control trials to observational studies and such comparisons may not be valid. Data on stool DNA tests was derived mainly from one study and this is subjected to bias. Second, while the analysis looked at differential adherence among screening tests, the possibility of decreasing adherence rates over time was not factored in. FIT is generally repeated every year, whereas the recommended interval for colonoscopy is ten years. It is likely that with frequent testing, adherence of FIT may decline over time. Third, the risk of CRC was not varied by socio-demographic status. This may be important because African-Americans have higher incidence of CRC and are more likely to have inadequate preps. Fourth, the model applies to a US population and setting, and may not be generalizable. The way screening programs are organized, funded and controlled are different in European countries and the US. The treatment protocols and costs can be significantly different in the US compared to single payor system in many European countries.”

In conclusion, this research shows that for colonoscopy to be a cost effective screening strategy for CRC, the inadequate bowel preparation rate should be less than 13%. For others, FIT may be the preferred screening strategy. Inadequate bowel preparation rates should be a reportable quality indicator.
